# Pharyngeal Microflora Disruption by Antibiotics Promotes Airway Hyperresponsiveness after Respiratory Syncytial Virus Infection

**DOI:** 10.1371/journal.pone.0041104

**Published:** 2012-07-26

**Authors:** Ke Ni, Simin Li, Qiuling Xia, Na Zang, Yu Deng, Xiaohong Xie, Zhengxiu Luo, Yan Luo, Lijia Wang, Zhou Fu, Enmei Liu

**Affiliations:** 1 Ministry of Education Key Laboratory of Child Development and Disorders, Key Laboratory of Pediatrics in Chongqing, Chongqing International Science and Technology Cooperation Center for Child Development and Disorders, Chongqing Medical University, Chongqing, China; 2 Department of Respiratory Medicine, Children’s Hospital of Chongqing Medical University, Chongqing, China; Louisiana State University Health Sciences Center, United States of America

## Abstract

**Background:**

Regulatory T cells (Treg cells), which are essential for regulation of immune response to respiratory syncytial virus (RSV) infection, are promoted by pharyngeal commensal pneumococcus. The effects of pharyngeal microflora disruption by antibiotics on airway responsiveness and relative immune responses after RSV infection have not been clarified.

**Methods:**

Female BALB/c mice (aged 3 weeks) were infected with RSV and then treated with either oral antibiotics or oral double distilled water (ddH_2_O) from 1 d post infection (pi). Changes in pharyngeal microflora were analyzed after antibiotic treatment for 7 d and 14 d. At 8 d pi and 15 d pi, the inflammatory cells in bronchoalveolar lavage fluid (BALF) were investigated in combination with tests of pulmonary histopathology, airway hyperresponsiveness (AHR), pulmonary and splenic Treg cells responses. Pulmonary Foxp3 mRNA expression, IL-10 and TGF-β1 in BALF and lung homogenate were investigated at 15 d pi. Ovalbumin (OVA) challenge was used to induce AHR after RSV infection.

**Results:**

The predominant pharyngeal commensal, *Streptococcus*, was cleared by antibiotic treatment for 7 d. Same change also existed after antibiotic treatment for 14 d. After RSV infection, AHR was promoted by antibiotic treatment at 15 d pi. Synchronous decreases of pulmonary Treg cells, Foxp3 mRNA and TGF-β1 were detected. Similar results were observed under OVA challenge.

**Conclusions:**

After RSV infection, antibiotic treatment cleared pharyngeal commensal bacteria such as *Streptococcus*, which consequently, might induce AHR and decrease pulmonary Treg cells.

## Introduction

Respiratory syncytial virus (RSV) is one of the most common pathogens responsible for lower respiratory tract infection in infants and young children. It causes bronchiolitis and is related to recurrent wheezing in later childhood [1]. However, the exact factor that links RSV infection and recurrent wheezing is not clear. Antibiotic use, resulting in alteration of bacterial colonization in airway in neonates, is related to later recurrent wheezing [2], [3]. Globally, antibiotic treatment is used frequently in patients with RSV infection [4]. However, antibiotic treatment is only recommended when bacterial infection is strongly suspected or proven after RSV infection [5], [6] and the rate of RSV and bacteria co-infection is not high [7], [8]. It is speculated that abuse of antibiotic treatment is the link between RSV infection and recurrent wheezing.

Wheezing is related to the differences of nasopharyngeal bacterial colonization [3]. In children with wheezing, bacterial colonization of the airway contains less dominant bacteria and more non-dominant bacteria than that of healthy children [9]. The pharyngeal microflora affects immune responses in surrounding lymphoid tissue. A higher proportion of Treg cells among adenoid mononuclear cells of children with positive pharyngeal commensal pneumococcus culture has been reported [10]. The whole cell antigen of pneumococcus induces adenoid IL-10-producing Treg cells proliferation [10]. Treg cells play an important role in modulating immune response to RSV infection. Increased Treg cells in peripheral blood or lung after RSV infection have been found in humans and mice [11]–[13]. The inhibitory effect of Treg cells on CD8+ T cells may balance RSV clearance and pathological processes and thus contribute to recovery [12]–[15].

Disruption of commensal microflora caused by antibiotic treatment reduces pulmonary Treg cells and induces more severe airway inflammation in response to aero-allergen ovalbumin (OVA) challenge [16]. It is not clear whether this effect links RSV infection and later wheezing. In this study, pharyngeal microflora was disrupted using the broad-spectrum antibiotics cefoperazone, to confirm its effect on RSV-induced airway inflammation, airway hyperresponsiveness (AHR) and to investigate the mechanisms involved. Previous studies indicate that following RSV infection, OVA sensitization via airway increases airway responsiveness to methacholine [17]. Therefore, OVA challenge was introduced in order to study the effect of disrupted pharyngeal microflora on airway responsiveness induced by both pathogen and allergen exposure. Our study shows for the first time that after RSV infection in early life, pharyngeal microflora disruption by antibiotic therapy can promote AHR and reduce pulmonary Treg cells synchronously.

## Materials and Methods

### Animal Model and Ethics Statement

Female BALB/c mice (10±2 g, aged 3 weeks) were housed under specific pathogen-free conditions in individual ventilated cages at the Laboratory Animal Center of Chongqing Medical University. Animals were provided with food and sterile water *ad libitum*. Treatment protocols were conducted as follows: (1) oral cefoperazone treatment *ad libitum* after RSV infection: contained the RSV+7d-Antibiotics group and the RSV+14d-Antibiotics group; (2) oral cefoperazone treatment *ad libitum* after mock infection: contained the 7d-Antibiotics group and the 14d-Antibiotics group; (3) oral ddH_2_O treatment *ad libitum* after RSV infection: contained RSV groups tested at 8 d pi and 15 d pi; (4) oral ddH_2_O treatment *ad libitum* after mock infection: contained mock groups tested at 8 d pi and 15 d pi. Changes in pharyngeal microflora were tested by polymerase chain reaction (PCR) at 8 d pi and 15 d pi. Airway inflammation, AHR, pulmonary and splenic Treg cells responses were analyzed in parallel. 1% (1 g/100 ml PBS) OVA aerosol challenge for 30 min/d from 8 d pi to 14 d pi was administrated to amplify AHR after RSV infection, and identical tests were conducted at 15 d pi. PBS challenge was used as control. All animals were treated in strict accordance to the guidelines for the Laboratory Animal Use and Care from Chinese CDC and the Rules for the Medical Laboratory Animal (1998) from the Ministry of Health, China, under the protocols approved by National Institute for Communicable Disease Control and Prevention. All actions on animals were approved by the Ethnics Committee of Chongqing Medical University.

### Virus

Hep-2 cells were cultured in DMEM medium with 10% fetal bovine serum (FBS). RSV A2 strain was grown in Hep-2 cells with DMEM medium supplemented with 2% FBS. The virus was purified as follows: after cells and supernatant were harvested, repeated freezing and thawing were carried out to fully release the virus, and then were centrifuged at 4°C. The supernatant was harvested as virus suspension and frozen at −80°C. It was adjusted to contain 3−5×10^7^ PFU of RSV/ml as assessed by plaque assay.

### Infection of Mice

Mice were infected under anesthesia by intranasal inoculation of RSV (10^6^ PFU in 100 µl virus suspension). Control mice were sham-infected with the DMEM medium with 2% FBS, which was centrifuged under the same conditions as the virus suspension’s. The efficacy of infection was certified by plague assay and PCR in whole lung sample [18], [19]. At 4 d pi, mice were sacrificed. The lungs were removed and partitioned, either (a) homogenized and cultured in Hep-2 cells, followed by examination of infection, demonstrated by the presence of syncytial lesions; or (b) used to extract RNA (Bioteke RNzol Reagent, Beijing, China) and reversely transcribed into cDNA (Takara PrimeScript RT reagent Kit, Dalian, China). RSV G protein gene primers were: P1: 5′-TGGGACACTCTTAATCAT-3′ and P2: 5′-TGATTCCAAGCTGAGG AT-3′. The PCR products were sequenced to verify RSV infection.

### Antibiotic Treatment and Identification of Pharyngeal Bacterium

As previously reported [20], Cefoperazone (0.5 mg/ml; Sigma-Aldrich, St. Louis, Mo. USA) was administered orally to mice *ad libitum* in drinking water. At 8 d pi and 15 d pi, some mice were randomly sacrificed. Throat swabs were cultured using Columbia Blood Agar, in an incubator with 5% CO_2_ for 20 h. Then, all colonies in the agar were collected and purified. The total DNA was extracted by DNeasy Kit (QIAGEN, Hilden, Germany). PCR of the V3 conservative region of 16s rRNA gene of all bacteria, and the specific region of *Streptococcus* and *E coli* were performed to confirm the antibiotic-induced disruption of pharyngeal microflora. Primers used were as follows: V3 F: 5′-CCTACGGGAGGCAGCAG-3′, R: 5′-ATTACCGC GGCTGCTGG-3′; *Streptococcus* F: 5′-GTACAGTTGCTTCAGGACGTATC-3′, R: 5′-ACGTTCGATTTCATCACGTTG-3′; *E coli*. F: 5′-CAATTTTCGTGTCCCCTTC G-3′, R: 5′-GTTAAT GATAGTGTGTCGAAAC-3′ [21]. All products were proved by sequencing. Counting of pharyngeal bacterial colonies was performed as follows: mouse was sacrificed, its pharyngeal mucosa tissue was removed and grinded in PBS at 100 mg: 1 ml. 10 µl of the pharyngeal mucosa tissue homogenate was cultured dispersedly on Columbia Blood Agar, in an incubator with 5% CO_2_ for 20 h. All bacterial colonies growing on Columbia Blood Agar were counted.

### Bronchoalveolar Lavage Fluid and Lung Homogenate Analysis

For bronchoalveolar lavage fluid (BALF) analysis, mouse was sacrificed and its airway was lavaged six times with 0.5 ml PBS, then BALF was centrifuged. The supernatant was collected and stored at −80°C to test IL-10 and TGF-β1 by use of ELISA kits (Beijing 4A Biotech Co., Ltd. China), whereas the cells at bottom were classified according to standard morphologic criteria in 100 cells via light microscopy after Wright-Giemsa staining. For lung homogenate analysis, mouse was sacrificed and its lung was removed and weighted. All the lung tissue was grinded in PBS at 100 mg: 1 ml, and homogenate was then centrifuged. The supernatant was collected and stored at −80°C to test IL-10 and TGF-β1 by use of ELISA kits (Beijing 4A Biotech Co., Ltd. China).

### Airway Responsiveness Detection

Pulmonary function measurement was performed by whole-body plethysmography (EMKA Technologies, Paris, France). AHR was expressed as enhanced pause (Penh) under each concentration of Methacholine (Sigma-Aldrich, St. Louis, Mo. USA), using PBS as baseline.

### Flow Cytometry Analysis

To test the Treg cells, lung and spleen were minced separately in PBS. Collagenase A was used to digest only the lung tissue. All cells passed through a 45 µm mesh, and were centrifuged at 450 g for 10 min at room temperature. Cells at the bottom were collected and washed, and the single-cell suspension was sent for flow cytometry analysis. Monoclonal antibodies anti-mouse CD4-FITC (Clone GK1.5, BD Biosciences, Heidelberg, Germany), anti-mouse CD25-PE (Clone PC61.5, eBioscience, SanDiego, USA) and anti-mouse Foxp3-PE-Cy5 (Clone FJK-16s, eBioscience, SanDiego, USA) were used to label the Treg cells. For intracellular staining of Foxp3, Foxp3 Fixation/Permeabilization (eBioscience, SanDiego, USA) and 10× Permeabilization (eBioscience, SanDiego, USA) were used. To prevent nonspecific binding of mAb, all samples were pre-incubated with purified rat IgG: anti-mouse CD16/CD32 monoclonal antibody (Clone 2.4G2, BD Biosciences, Heidelberg, Germany). Cellular phenotypes were measured using a BD FacsCaliber flow cytometer and analysis was carried out using the BD CellQuest software.

### Real Time - Polymerase Chain Reaction

RNA from lung tissues was extracted and reversely transcribed into cDNA. The Foxp3 and β-actin were quantified by Real-time PCR (BIO-RAD, Hercules, USA). SYBR Green RealMasterMix (TIANGEN, Beijing, China) as well as the following primers were used as fluorescence: Foxp3: F: 5′-AGCTGGAGCTGGAAAAGGA-3′ and R: 5′-GCTACGATGCAGCAAGAGC-3′; β-actin F: 5′-TGGCATTGTTACCA ACTGGGAC-3′ and R: 5′-TCACGGTTGGCCTTAGGGTTC-3′. Both targets were run in triplicate.

**Figure 1:**
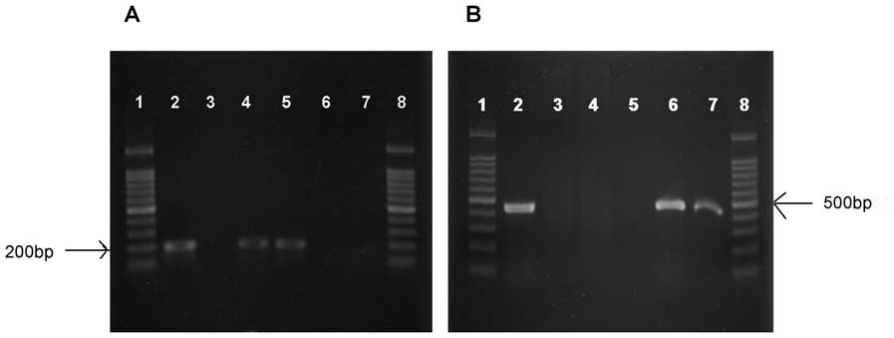
Examination of pharyngeal microflora. (A) PCR amplification of the specific region of *Streptococcus*. 1, Marker; 2, Positive control, streptococcus NCTC7466; 3, Negative control; 4, No RSV infection; oral ddH2O for 7 d; 5, After RSV infection, oral ddH2O for 7 d; 6, No RSV infection, oral antibiotics for 7 d; 7, After RSV infection, oral antibiotics for 7 d; 8, Marker. PCR product, 197 bps. (B) PCR amplification of the specific region of *E. coli*. 1, Marker; 2, Positive control, *E. coli* ATCC25922; 3, Negative control; 4, No RSV infection, oral ddH2O for 7 d; 5, After RSV infection, oral ddH2O for 7 d; 6, No RSV infection, oral antibiotics for 7 d; 7, After RSV infection, oral antibiotics for 7 d; 8, Marker. PCR product, 450 bps.

### Statistical Analysis

Graphpad Prism 5.0 utilizing Two-way ANOVA with Bonferroni post-tests were used to analyze AHR and inflammatory cell analysis. Other data were compared using Kruskal-Wallis test. All experimental results were expressed as mean ± SEM and *P*<0.05 was considered to be statistically significant.

**Figure 2:**
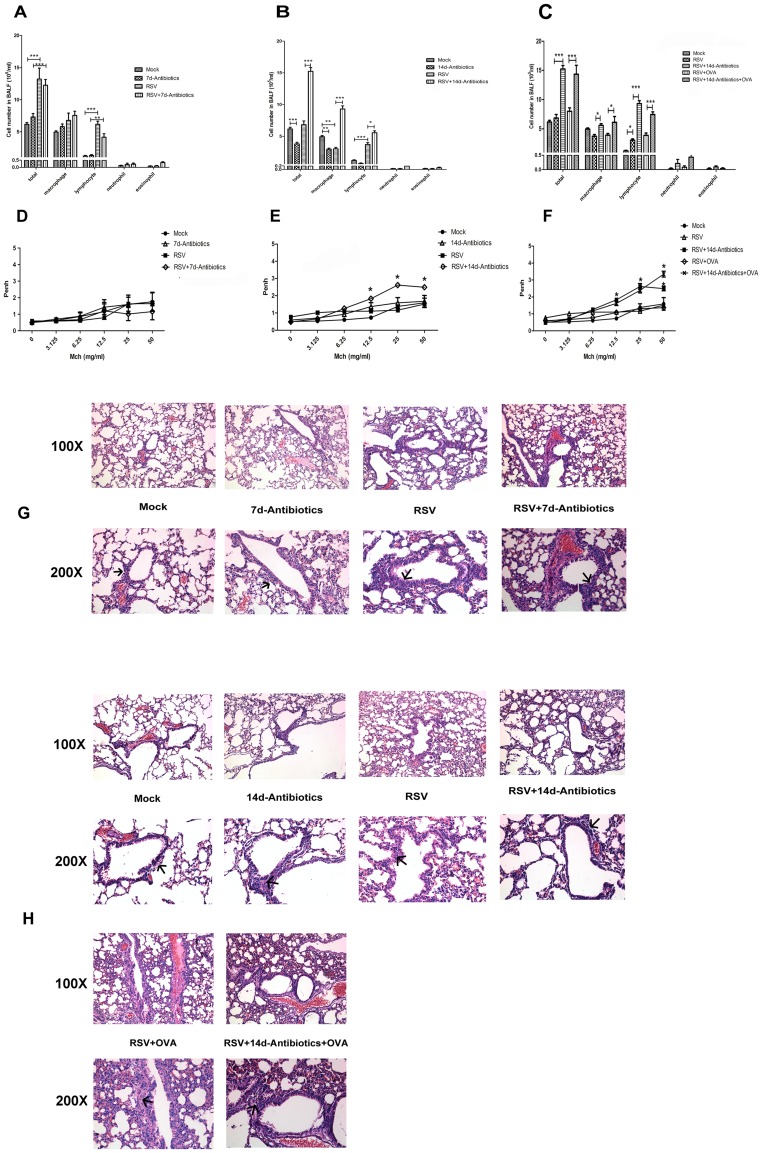
Airway inflammation, hyperresponsiveness analysis and histopathological analysis. (A) Inflammatory cell counts and classification in BALF at 8 d pi. (B) Inflammatory cell counts and classification in BALF at 15 d pi. (C) Inflammatory cell counts and classification in BALF at 15 d pi. OVA exposure was introduced. (D) Airway hyperresponsiveness analysis at 8 d pi. (E) Airway hyperresponsiveness analysis at 15 d pi. (F) Airway hyperresponsiveness analysis at 15 d pi. OVA exposure was introduced. (H) Histopathological analysis (H&E, 100× and 200×) of the lung at 8 d pi. Infiltrating inflammatory cells are marked by arrow. (G) Histopathological analysis (H&E, 100× and 200×) of the lung at 15 d pi. Infiltrating inflammatory cells are marked by arrow. Data (N  = 5−6) are presented as the mean ± SEM of three independent experiments. Please note that some groups of data are presented twice in different panels for better interpretation. *P<0.05, **P<0.01, ***P<0.001.

**Figure 3:**
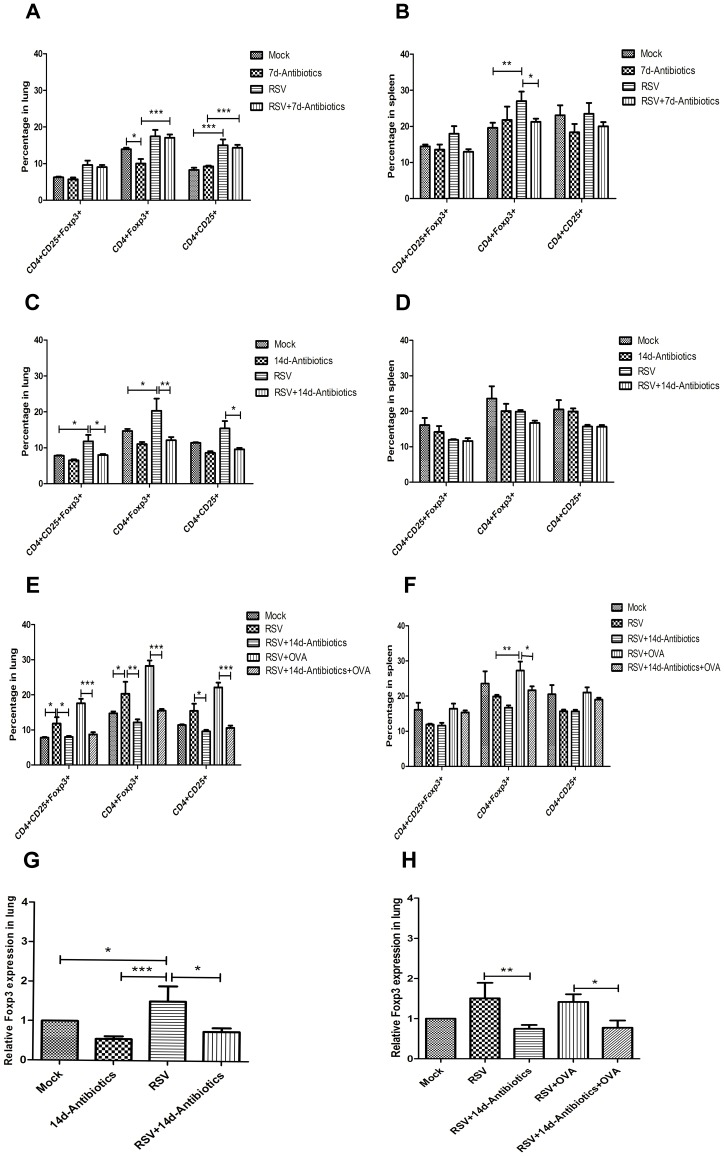
Flow cytometry analysis of Treg cells response and real time-PCR analysis of Foxp3 mRNA expression. (A) Percentages of pulmonary Treg cells at 8 d pi. (B) Percentages of splenic Treg cells at 8 d pi. (C) Percentages of pulmonary Treg cells at 15 d pi. (D) Percentages of splenic Treg cells at 15 d pi. (E) Percentages of pulmonary Treg cells at 15 d pi. OVA exposure was introduced. (F) Percentages of splenic Treg cells at 15 d pi. OVA exposure was introduced. (G) Relative Foxp3 mRNA expression in lung at 15 d pi. The mock treated group was used as the standard. (H) Relative Foxp3 mRNA expression in lung at 15 d pi, OVA exposure was introduced. The mock treated group was used as the standard. Data (N  = 5−6) of FCM are presented as the mean ± SEM of three independent experiments. Please note that some groups of data are presented twice in different panels for better interpretation. Data of each group in mRNA expression analysis are based on N  = 7−8. * *P*<0.05, **P<0.01, ***P<0.001.

**Figure 4:**
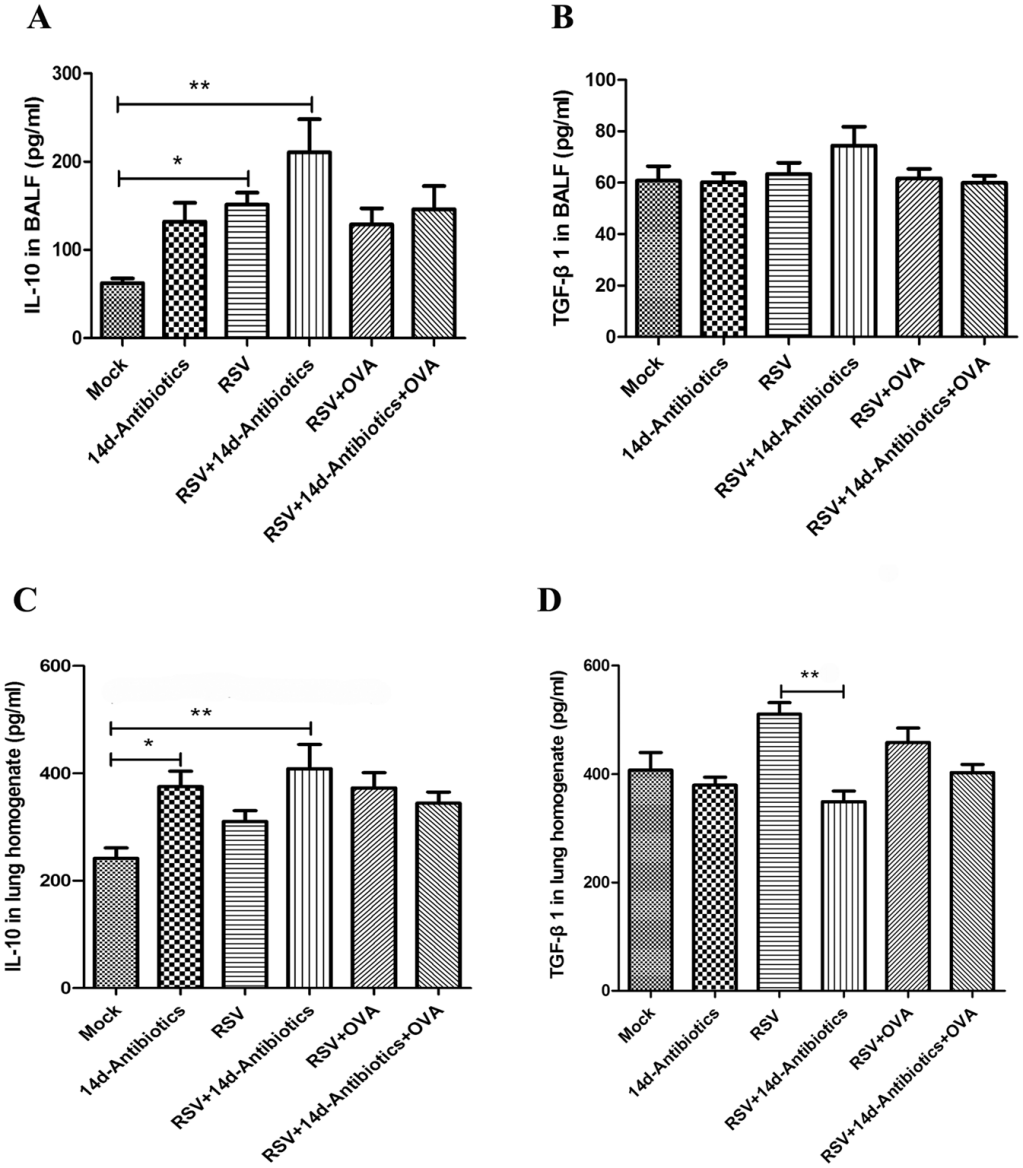
ELISA analysis of IL-10 and TGF-β1 in BALF. (A) IL-10 expression in BALF. (B) TGF-β1 expression in BALF. (C) IL-10 expression in homogenate. (D) TGF-β1 expression in homogenate. Data (N  = 7−9) are presented as the mean ± SEM of two independent experiments. * *P*<0.05, **P<0.01, ***P<0.001.

## Results

### RSV Infection in Mice

Mice infected with RSV were sacrificed at 4 d pi. RSV titers in the lung were analyzed by plaque assay as 60.68±11.71 PFU/ml lung homogenate. RSV infection was also verified by PCR and sequencing.

### Cefoperazone Disrupts the Pharyngeal Microflora in Mice

Without treatment, the predominant pharyngeal commensal bacterium was *Streptococcus,* as previously described [22]. After cefoperazone treatment for 7 d, *Streptococcus* in pharyngeal mucosa of mice was cleared (Fig. 1A), whereas some resistant *E coli* were detectable (Fig. 1B). The number of bacterial colonies after cefoperazone treatment (3.00±0.58×10^3^ CFU/g of pharyngeal mucosa tissue) was markedly lower than the number (127.30±5.13×10^3^ CFU/g of pharyngeal mucosa tissue) in mock group. RSV infection did not affect the predominant microflora according to PCR analysis (Fig. 1A, 1B). At 15 d pi, the same change of pharyngeal microflora was found (data not shown).

### Pharyngeal Microflora Disruption by Antibiotics Promotes AHR after RSV Infection

Enumeration of inflammatory cells in BALF, histological analysis and AHR tests were performed at 8 d pi and 15 d pi (Fig. 2A–H). RSV infection induced an obvious increase of inflammatory cells in BALF at 8 d pi but not at 15 d pi (Fig. 2A, 2B). Significant differences in subgroups of these inflammatory cells were detected. Normally, macrophages predominated in BALF (Fig. 2A, 2B). Increased number of lymphocytes was observed in BALF at 8 d pi and 15 d pi, whereas the number of macrophages did not increased highly and even reduced at 15 d pi (Fig. 2A, 2B). RSV infection alone did not induce obvious AHR at these time-points (Fig. 2D, 2E). Histopathological analysis revealed, after RSV infection, more inflammatory cell infiltrated around the bronchi at 8 d pi (Fig. 2G) and 15 d pi (Fig. 2H), although this injury was reduced at 15 d pi compared with 8 d pi. Antibiotic treatment after RSV infection also resulted in accumulation of inflammatory cells in airway, again predominantly lymphocytes, at 8 d pi (Fig. 2A) and 15 d pi (Fig. 2B). Antibiotic treatment after RSV infection induced AHR in response to methacholine challenge at high concentrations at 15 d pi but not at 8 d pi (Fig. 2D, 2E). Histopathological injury in the RSV plus antibiotic treatment group did not show significant difference from that of the RSV infection groups at these time-points (Fig. 2G, 2H). Antibiotic treatment alone did not obviously influence the inflammatory cell count in BALF, histopathological injury or AHR compared with mock group (Fig. 2A–H). In response to OVA challenge, no deteriorative trends were observed in inflammatory cell count in BALF or AHR tests (Fig. 2C, 2F), although greater inflammatory cell infiltration was found around the bronchi (Fig. 2H).

### Pharyngeal Microflora Disruption by Antibiotics Results in a Decrease in Pulmonary Treg Cells and Foxp3 mRNA Expression after RSV Infection

To investigate changes in Treg cells, their percentages were tested synchronously in lung and spleen. At 8 d pi, no obvious differences were detected among all groups. RSV infection increased pulmonary CD4+CD25+ cells and splenic CD4+Foxp3+ cells (Fig. 3A, 3B). Antibiotic treatment alone reduced CD4+Foxp3+ cells in lung (Fig. 3A). After RSV infection, antibiotic treatment reduced CD4+Foxp3+ cells in spleen but not in lung (Fig. 3A, 3B) compared with that of RSV infection alone. At 15 d pi, obvious changes were observed in lung. After RSV infection, CD4+CD25+Foxp3+ cells increased in lung although, interestingly, the increase of CD4+CD25+Foxp3+ cells was inhibited in the RSV infection plus antibiotic treatment group (Fig. 3C). Antibiotic treatment alone also inhibited pulmonary CD4+CD25+Foxp3+ cells, although this effect did not reach the level of statistical significance. The differences in spleen were not obvious (Fig. 3D). In response to OVA challenge, this antibiotic inhibition phenomenon was also observed in lung and spleen (Fig. 3E, 3F). Similar trends were also observed in pulmonary Foxp3 mRNA expression analyses (Fig. 3G, 3H).

### Pharyngeal Microflora Disruption by Antibiotics Decreases TGF-β1 in Lung Homogenate after RSV Infection

IL-10 and TGF-β1 in BALF and lung homogenate were tested at 15 d pi. In BALF, RSV infection increased IL-10 expression. In contrast to pulmonary Treg cells change, antibiotic treatment after RSV infection led to increased IL-10 expression in BALF (Fig. 4A), No obvious changes in TGF-β1 expression were observed among different groups (Fig. 4B) in BALF. In lung homogenate, both of only antibiotic treatment and RSV infection plus antibiotic treatment could increase IL-10 level (Fig. 4C), whereas antibiotic treatment after RSV infection reduced TGF-β1 level compared with that of only RSV infection group (Fig. 4D).

## Discussion

RSV is the most common pathogen associated with risk of wheezing in infants and young children. Stein RT, et al. find that bronchiolitis caused by RSV in early life is associated with an increased risk of wheezing later on [23]. Other research identifies that children hospitalized for bronchiolitis during infancy have more frequent wheezing episodes and follow-up visits for asthma at seven years of age comparing with age-matched controls [24]. However, not all infants with bronchiolitis will develop subsequent recurrent wheezing. The trigger factors are complex and not clearly understood. The findings of our studies indicated that disruption of pharyngeal microflora by overuse of broad-spectrum antibiotics following RSV infection in early life might trigger AHR. To our best knowledge, this is the first study addressing the effect of pharyngeal microflora on airway inflammation and subsequent AHR following RSV infection.

Firstly, we demonstrated the disruption of the pharyngeal microflora following oral antibiotic treatment. Under normal conditions, *Streptococcus* was found to be the predominant pharyngeal bacterium, which was in accordance with previous studies [22]. It could be speculated that rapid growth of the predominant *Streptococcus* in the limited space competitively inhibited the growth of the non-dominant *E coli.* Therefore, *Streptococcus* was positive whereas *E coli* was negative in non-antibiotic treated groups. Following antibiotic treatment, *Streptococcus* was cleared while antibiotic-resistant *E coli* remained. It removed the space constraint on *E coli* growth. Consequently, *Streptococcus* was negative whereas *E coli* was positive in the antibiotic treated groups.

Secondly, we demonstrated pharyngeal microflora disruption by antibiotics promoted AHR after RSV infection. In our study, RSV infection induced greater inflammatory cell accumulation in BALF and infiltration around the bronchi at 8 d pi. Furthermore, after period of acute inflammation, the inflammation relieved relatively at 15 d pi. However, AHR was not detected at these time-points. In different studies, the persistent time of AHR after RSV infection varies. [25], [26]. AHR Peak can be detected during the first 6 days after RSV infection, which is followed by a rapid reduction in this effect. In this study, AHR tests were performed at 8 d pi and 15 d pi, which were outside this peak period. This is considered to be the reason for the absence of AHR in our RSV infection model. Actually, we found AHR peak appeared at 4 d pi, reduced at 6 d pi and returned to normal level at 8 d pi (data not shown). However, when pharyngeal microflora was disrupted by antibiotic treatment for 14 days after infection, AHR was significantly induced at 15 d pi, thus indicating the effect of pharyngeal microflora on airway responsiveness.

The relationship between bacterial colonization in neonates and recurrent wheezing in later childhood has been reported. The Copenhagen prospective study on asthma in a childhood cohort indicates that neonates colonized with specific bacteria in the hypopharyngeal region are at increased risk of recurrent wheeze and asthma in early life [3]. Bacterial colonizations in airways are different between children with recurrent wheezing and healthy children [9]. There are increasing evidences indicating that microflora disruption caused by antibiotics, accompanied by recurrent allergen challenge, disrupts the immune balance of airways and aggravates airway inflammation [16], [20]. In these studies, all of airway inflammation and AHR were allergen-induced. Our research is the first to demonstrate the influence of pharyngeal commensal microflora on pathogen-induced airway inflammation and AHR.

Thirdly, we observed that pharyngeal microflora disruption by antibiotics resulted in a decrease in Treg cells and Foxp3 mRNA expression in the lung after RSV infection. The search for a Treg cells-specific molecular marker has revealed that the majority, if not all, of these cells constitutively express the CD25 molecule and depletion of CD25+CD4+ T cells spontaneously evokes autoimmune disease [27]. Recent efforts have shown that natural Treg cells specifically express the transcription factor Foxp3 and that mutations of the Foxp3 gene produce a variety of immunological diseases in humans [28]. In our study, frequencies of CD4+CD25+Foxp3+, CD4+Foxp3+ and CD4+CD25+ cells were analyzed. RSV infection induces Treg cells, which has been widely reported in human and animal studies [11]–[13]. In our study, after RSV infection, pulmonary Treg cells increased significantly at 15 d pi. Pulmonary Treg cells were not fully activated during the early adaptive immune period at 8 d pi, which might explain the absence of an increase at 8 d pi. The inhibitory effect of antibiotic treatment for 14 d on pulmonary Treg cells was confirmed by flow cytometry and Foxp3 mRNA expression test. Moreover, in the antibiotic treatment alone group, the inhibitory effect was predominantly focused on CD4+Foxp3+ cells. Our results are in accordance with previous studies that shows a lower relative gene expression of Foxp3 in germ-free mice [29] and a high frequency of adenoid Treg cells in children with positive nasopharyngeal pneumococcus culture, which indicate the role of pharyngeal commensal microflora in maintaining immune homeostasis via Treg cells proliferation [10]. These studies provide clear evidence of the influence of commensal colonization on immune tolerance mediated via Treg cells. Commensal microflora promotes the proliferation of Treg cells [10], [30], [31]. In our study, antibiotic treatment significantly reduced pharyngeal commensal microflora, therefore prohibited the stimulation on Treg cells proliferation. The response of splenic Treg cells differed from the pulmonary one, which indicated that the influence of pharyngeal microflora disruption was constrained within local tissues.

Most interestingly in our study, the direction of pulmonary Treg cells change was in contrast to that of AHR change. This finding is consistent with previous study showing opposition of the Treg cells response compares with changes in AHR induced by allergen [32]. Furthermore, attenuation of AHR mediated by airway Treg cells has been confirmed by adoptive transfer of Treg cells into mice [33]. Differently, AHR was induced by pathogen infection in our study.

Finally, IL-10 and TGF-β1 level in BALF and lung homogenate were tested. IL-10 and TGF-β1 are considered to be effector cytokines of peripheral Treg cells. Treg cells that co-express TGF-β1 and IL-10 enable suppression of allergen-induced AHR [33]. In our study, the increased IL-10 in BALF and lung homogenate was observed in RSV infection plus antibiotic treatment group at 15 d pi, which seemed to be contradictory to the change of pulmonary Treg cells. In fact, IL-10 is also produced by Th2 cells [34]. The IL-10 deriving from Treg cells in BALF and lung homogenate can hardly be tested by ELISA, so another precise method is required. On the other hand, the decreased TGF-β1 in lung homogenate was observed in RSV infection plus antibiotic treatment group at 15 d pi, which was in parallel with the decrease in pulmonary Treg cells. TGF-β1 can be produced by lymphocytes, macrophages and dendritic cells [35]. Studies on TGF-β1 and AHR indicate the increased AHR is related to decreased expression of TGF-β1 and Foxp3 in the lung [36] and TGF-β1 suppresses airway hyperresponsiveness in allergic airway disease [37]. TGF-β1 also suppresses parainfluenza virus-induced AHR [38]. Our study also found the higher AHR when 14d-antibiotic treatment reduced pulmonary TGF-β1 after RSV infection. TGF-β1 in BALF did not show any change. According to our consideration, the low level of TGF-β1 in BALF (only about 60 pg/ml) may hide the differences among each group.

In conclusion, our study indicated that antibiotic treatment disrupted pharyngeal microflora and eliminated its capacity to promote Treg cells proliferation and activity, subsequently promoted AHR in response to RSV infection. The lymphoid tissues located in the mucosa and the mucosa-associated lymphoid tissue (MALT) are the first sites to contact with the commensal flora [39]. The typical MALT in the respiratory tract contains nasal-associated lymphoid tissues (NALT) and bronchi-associated lymphoid tissues (BALT), both of which comprise the cell composition expected in an immune inductive site, and have the capacity to respond to airway antigens [40]. This may provide an immune inductive site for the surrounding microflora. Antibiotic treatment after RSV infection will disrupt the pharyngeal microflora, potentially influence the local immune response and thereby lead to AHR. Cautious use of antibiotics following RSV infection is therefore warranted.
